# Apexification with Calcium Hydroxide vs. Revascularization

**DOI:** 10.1155/2020/9861609

**Published:** 2020-05-27

**Authors:** H. Boufdil, M. Mtalsi, S. El Arabi, B. Bousfiha

**Affiliations:** Department of Pediatric Dentistry, Faculty of Dentistry, Hassan II University of Casablanca, Morocco

## Abstract

Treatment of necrotic immature teeth has always been a real challenge for the clinician due to the open apex and weak root structure, which does not allow a conventional endodontic treatment. Several therapeutic options are possible to treat those teeth. Calcium hydroxide apexification is the oldest and most studied therapeutic option, but it has some disadvantages, including the long term of treatment, the possibility of reinfection, and the weakness of the wall. To solve these problems, several authors recommend the revascularization technique. This therapy allows the continuation of root edification with apical closure and thickening of the walls. The aim of our work is to compare the two therapeutic procedures, apexification with calcium hydroxide and revascularization, through a clinical case treated and followed up for 24 months at the pediatric dentistry department in Casablanca, Morocco.

## 1. Introduction

Dental trauma injuries account for 30% of pulpal necrosis on an immature permanent tooth [1]. It is difficult to get an appropriate apical seal in teeth with open apices by using the conventional endodontic treatment methods. The discontinued development of dentinal walls after the pulp necrosis can also lead to a weak root structure with thin dentinal walls, which makes the tooth susceptible to future fractures [2].

For decades, the apexification technique with calcium hydroxide has proven its effectiveness in solving these problems. It is the most studied and less expensive technique to induce a calcified apical barrier with a success rate of 74% [2] to 100% [3]. However, this technique, which is considered a long-term procedure (6 to 24 months to obtain the apical barrier), does not increase the thickness or length of the root wall and make the tooth fragile and susceptible to future fractures.

An alternative technique using MTA has progressively replaced the apexification with calcium hydroxide. The main quality of this material is to reduce the number of sessions to create an apical barrier. Nevertheless, this technique remains very expensive and shares the same disadvantages with calcium hydroxide of not allowing the continuation of root development, which leads to a fragile root structure.

Recently, scientific research had led to the development of a new concept called revascularization to treat necrotic immature permanent teeth. This technique allows the continuation of root edification while the periapical lesion is healing. However, this protocol is not sufficiently studied and required a certain clinical dexterity. The origin and the nature of the tissue formed in the canal are still controversial. Several authors performed a histological study on humans' and animals' tooth.

Another concept called pulp regeneration has emerged. This technique uses a growth factor and the regenerative capacity of the pulpodentinal complex from pulp stem cells [4]. The growth factors will induce cell proliferation and differentiation as well as the migration of progenitor cells.

The aim of this report is to compare two therapeutic procedures: apexification with calcium hydroxide and pulp revascularization though a clinical case with over 24 months of follow-up in the pediatric dentistry department in Casablanca.

## 2. Clinical Case

A healthy 7-year-old boy was referred from the dental emergency department to the pediatric dentistry department in the Dental School of Casablanca.

**Figure 1:**
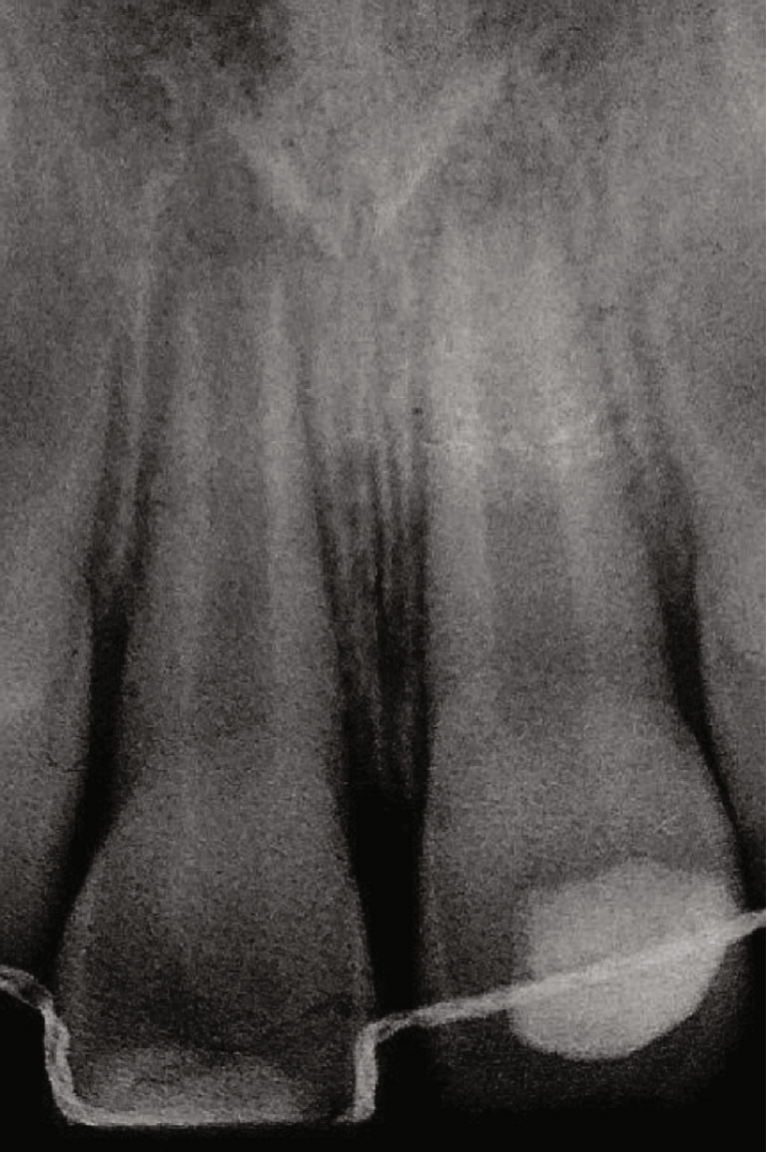
Retroalveolar radiograph. The tooth had immature roots (Nolla's stage 8).

Clinical examination revealed that the child has a flexible splint on maxillary teeth and a coronal restoration with a glass ionomer on 11. Radiographic examination showed incomplete root formation of both central incisors (Nolla's stage 8: short root with thin walls) (Figure 1).

Based on the emergency file, it was found that the boy fell at school three weeks ago. Clinical examination revealed that 11 had a lateral luxation with an uncomplicated crown fracture and 21 had a subluxation. Emergency treatment was carried out on the same day and consisted of reattaching the tooth fragment, repositioning 11 with a gentle digital pressure, and stabilizing it with a flexible splint. One week after trauma, the patient presented with intraoral swelling on 11 revealing necrosis. Consequently, an endodontic treatment was performed and the root canal was instrumented using hand files with gentle irrigation using 0.5% sodium hypochlorite. Calcium hydroxide was placed as intracanal medication. The access cavity was sealed with glass ionomer cement.

**Figure 2:**
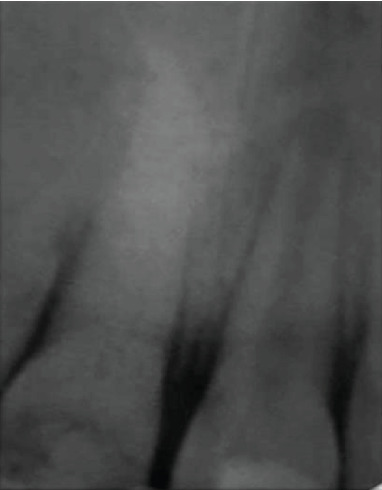
Retroalveolar radiograph showing the calcium hydroxide filling of 11.

For this purpose, the apexification technique using calcium hydroxide has been initiated on 11. The calcium hydroxide was renewed once resorbed. A regular clinical and radiological follow-up was carried out to check the vitality of 21 (Figure 2).

**Figure 3:**
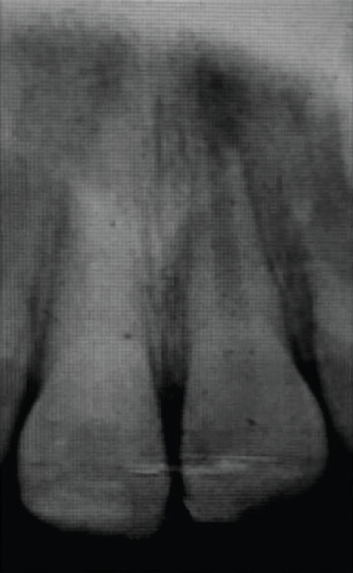
6-month follow-up radiograph: a radiolucent periapical lesion adjacent to 21. The negative response to the cold test confirms pulp necrosis.

6 months after the beginning of the treatment of 11, a radiolucent lesion in the periapical region was detected on 21 (Figure 3). The negative response to the cold test confirms a pulp necrosis. Considering the immaturity of the tooth and the thinness of the root and after obtaining parental consent, the optimal treatment option was revascularization.

An access cavity was made under a dental dam. Once the canal orifice was well cleared, gentle root canal irrigation was performed with 2% sodium hypochlorite followed by irrigation with saline solution. Subsequently, the root canal was dried with large paper points until they were removed without evidence of fluids and filled with calcium hydroxide used as intracanal medication. A temporary restoration using a glass ionomer was placed.

**Figure 4:**
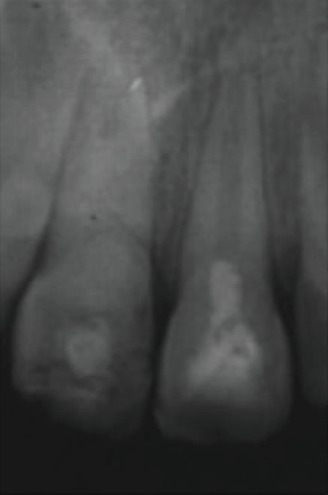
Retroalveolar radiograph showing capping of the blood clot with MTA.

The patient was recalled 4 weeks after the first appointment. The tooth was clinically and radiologically asymptomatic. In the second session, the tooth was anaesthetized, accessed, and irrigated gently with 2% sodium hypochlorite followed by saline solution to remove calcium hydroxide. The bleeding was induced with manual K files introduced into the root canal and placed at 2 mm beyond the working length. Once the blood clot was formed, MTA was placed in the cervical third of the root canal (Figure 4). The access cavity was sealed with a glass ionomer.

**Figure 5:**
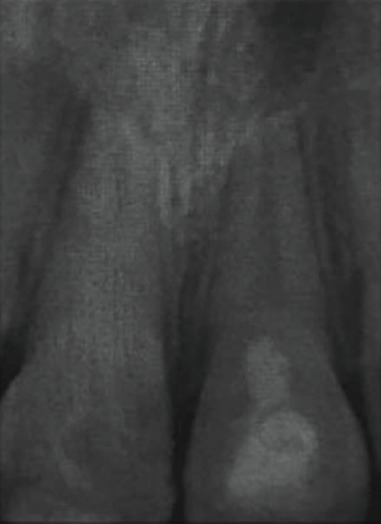
3-month follow-up radiograph after revascularization: beginning of root edification on 21.

**Figure 6:**
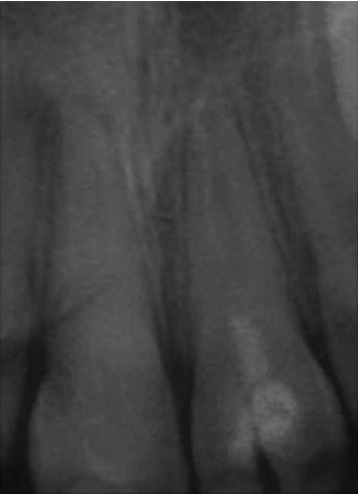
6-month follow-up radiograph after revascularization: thickening of the wall with the beginning of root calcification on 21.

Three months after performing revascularization treatment, visible root edification was observed (Figure 5). The 6-month follow-up showed an important thickening of the root with calcification of the canal. Concerning 11, a renewal of the calcium hydroxide was carried out once the material has been absorbed (Figure 6).

**Figure 7:**
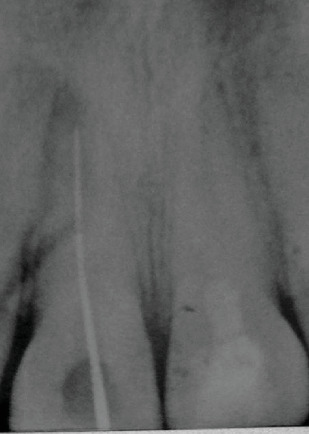
24-month follow-up radiograph of 11: an apical barrier was formed easily crossable with a file.

**Figure 8:**
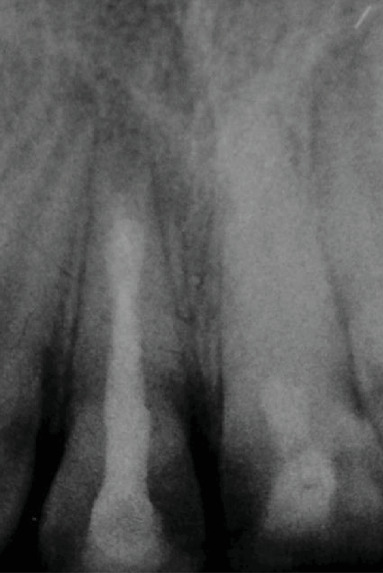
Root canal plugging of 11: MTA+gutta. Complete root canal obliteration of 21.

After 24 months of apexification treatment on 11, we noticed the formation of an apical barrier which was easily crossable with a file (Figure 7). To solve that problem, the apical third was sealed using MTA and then the coronal two-third with warm gutta-percha. Concerning 21, the canal was almost completely obliterated with an extension of the root (Figure 8).

## 3. Discussion

The completion of root development and closure of the apex occurs up to 3 years after the eruption of the tooth [5]. During this period, the tooth is exposed to several forms of aggression. According to Andreasen et al., 30% of pulp necrosis on immature permanent teeth is a consequence of dental trauma [1]. The crown fracture and the periodontal tissue injuries were responsible for the pulp necrosis in the clinical case.

Due to their anatomical complexities, the treatment of immature necrotic teeth remains a challenge for the clinician [6]. The achievement of a conventional endodontic treatment is impossible due to the open apex and the weak root structure.

To solve these problems, several therapeutic solutions have been described; the oldest one was the apexification. The American Association of Endodontists defined this technique as “a method to induce a calcified barrier in a root with an open apex or the continued apical development of an incomplete root in teeth with necrotic pulp” [7]. This calcified barrier is induced by several materials including calcium hydroxide. Indeed, this material has a potential to induce the osteodentin barrier formation. However, this therapeutic procedure has many inconveniences such as the long-term obtainment of the apical barrier (6 to 24 months), recontamination of the root canal system during treatment periods, a high risk of root fractures, and also the weakness of the apical barrier [8]. In the previous clinical case, some disadvantages were noticed during apexification treatment on 11. After 24-month treatment, an apical barrier easily crossed by a file was observed. Creating an artificial apical barrier with MTA was undertaken.

As early as the 1990s, a new apexification technique was adopted replacing calcium hydroxide with MTA (mineral trioxide aggregate). MTA is a powder consisting of fine hydrophilic particles of tricalcium silicate, tricalcium oxide, and silicate oxide. It has low solubility and a radiopacity that is slightly greater than that of the dentin [5]. The alternative to MTA had the advantage over the previous technique to reduce the number of clinical sessions and to produce apical hard tissue with significantly greater consistency but does not allow the root edification, and the root fragility persists [9].

Since the 21st century, the creation of a biological barrier by exploiting the potential of stem cell differentiation has been possible. This technique is commonly known as “revascularization.” Unlike apexification, this new technique allows the continuation of root development using the patient's stem cells [8]. However, this therapeutic procedure has not yet been sufficiently studied.

The origin and the nature of the tissue formed in the canal are still controversial. According to Moreno-Hidalgo et al. [10], a cellular microenvironment is generated from the vital tissue of the periapical region. These cells migrate through the blood clot into the pulp space. They are immature and multipotent and able to secrete hard tissue inside the canal. Other studies have shown that periodontal stem cells proliferate inside the canal and may be the origin of the formation of hard tissue into the canal [11]. Some authors suspected the presence of vital pulp cells that could be the origin of the neoformed tissue [3].

A histological study was performed on a tooth previously treated with the revascularization technique, and the tooth was extracted 3 weeks after the treatment in order to examine the nature of tissue formed in the root canal. The study showed that one-half of the canal had newly formed tissue similar to pulp tissue [12].

The success of pulp revascularization depends on three key factors: root canal disinfection, quality of blood clot, and hermetic root canal filling [13].

The removal of necrotic microorganisms and pulp tissue from the root canal is a critical factor for the success of the process [14]. Thus, the clinician is confronted with a wide option of root canal disinfection materials; the most commonly used is sodium hypochlorite. Thanks to its bactericidal effect, sodium hypochlorite is a material of choice for root canal disinfection. However, in order to limit the cytotoxicity, it is recommended that it should be used with a specific concentration (1.5% to 2%). The use of sodium hypochlorite followed by 17% of ethylenediaminetetraacetic acid (EDTA) irrigation allows better wettability of the irrigator and removal of the smear layer [15]. In our case, gentle irrigation with 2% of sodium hypochlorite was carried out in 21 before using calcium hydroxide as intracanal medication.

Besides irrigation products, intracanal medication has proven to be effective against infection. In 1996, Hoshino proposed a tri-antibiotic paste composed of metronidazole (500 mg), ciproloxacin (200 mg), and minocycline (100 mg). This paste has the ability to eliminate Gram +, Gram –, and anaerobic bacteria [16]. However, the use of this paste at high concentrations may have undesirable results on stem cells [17].

Considering the problem of dyschromia and storage constraints of the tri-antibiotic paste, we used calcium hydroxide as intracanal medication. Calcium hydroxide is widely used in endodontic treatment due to the chemical and antiseptic action. It disinfects the canal with maximum stem cell survival [18]. This dyschromia was well observed in 21.

The success of revascularization is closely linked to the formation of the blood clot. It is done with an endodontic instrument (K or H file) that goes beyond the apical area of a disinfected tooth. A lack of bleeding leads to a lack of root development [19].

The placement of a scaffold is done once the blood clot is stable. The role of these products is fundamental for the success of the treatment. It preserves the function and the vitality of the tissue regenerated [20]. Among these materials, there is MTA which, due to its physical properties, has proven its effectiveness. However, MTA did not show any antimicrobial properties against anaerobic species like *S. mutans* and *E. faecalis* or the strict anaerobic species like *P. gingivalis*, *P. intermedia*, and *F. nucleatum* [21]. Also, endodontic revitalization procedures using MTA are technically challenging and are often associated with tooth discoloration [22]. Since the 2000s, the marketing of Biodentine has solved the disadvantages of MTA. However, MTA has been extensively studied compared to Biodentine [4].

Some clinical studies have shown the effectiveness of revascularization to treat immature permanent teeth. In 2011, Nosrat et al. noticed an increase in root length in all three immature permanent teeth treated by the revascularization technique with a clinical follow-up of 20 months [8].

Another clinical study conducted by Cehreli et al. on six immature permanent teeth with a clinical follow-up of 10 months showed root development and wall thickening with formation of hard tissue on all teeth [23]. The same results were obtained in Reynolds et al.'s study on five immature permanent teeth with an apical lesion. A 24-month clinical and radiological follow-up revealed a significant decrease in the lesion (60% of treated teeth) [24].

In 2013, Chen et al. noticed a thickness of the wall in 81% of the treated teeth with a decrease in the apical lesion in all the teeth [25].

Radiographically in our case, the revascularization technique appears to show continued development by the deposition of hard tissue on the canal walls and continued root development.

The selection factor between apexification with calcium hydroxide and revascularization depends on several parameters. Belli et al. in 2017 demonstrated that the revascularization technique was more interesting biomechanically than the apexification technique [26]. Indeed, the risk of a root fracture of immature permanent teeth is related to lack of root growth [27, 28].

Other authors recommend revascularization for immature permanent teeth with a diameter superior to 1.1 mm. Indeed, the narrower the apical foramen is, the more restricted the blood flow is [29, 30].

However, for a therapeutic gradient logic, it would be interesting to proceed with the first intention to use a least invasive technique, namely, revascularization [28]. In case of failure, an apexification treatment can be considered.

Infection and inflammation of the tooth is also a decisive parameter for our therapeutic choice: hypothetically, the longer an infection exists, the lower the probability that pulp and stem cells required for regeneration will survive [31].

## 4. Conclusion

In conclusion, the present case shows that apexification and revascularization, despite being two different protocols with different results, have a common objective of treating the necrotic immature permanent tooth. Indeed, the therapeutic decision depends on the clinical situation, the desired results of the treatment, and the performance and dexterity of the dentist.
